# Minimal Effective Dose of Beans Required to Elicit a Significantly Lower Glycemic Response Than Commonly Consumed Starchy Foods: Predictions Based on In Vitro Digestion and Carbohydrate Analysis

**DOI:** 10.3390/nu15214495

**Published:** 2023-10-24

**Authors:** D. Dan Ramdath, Simone Renwick, Aileen Hawke, Davin G. Ramdath, Thomas M. S. Wolever

**Affiliations:** 1 Guelph Research and Development Centre, Agriculture and Agri Food Canada, Guelph, ON N1G 5C9, Canada; srenwick@health.ucsd.edu (S.R.); aileen.hawke@agr.gc.ca (A.H.); davin.ramdath@mail.utoronto.ca (D.G.R.); 2 Inquis Clinical Research, Toronto, ON M5C 2N8, Canada

**Keywords:** beans, glucose response, carbohydrates, minimum effective dose

## Abstract

Beans elicit lower glycemic responses (GRs) than other starchy foods, but the minimum effective dose (MED) to reduce GR is unknown. We sought to determine the MED of beans compared to common starchy foods. Overnight-fasted healthy volunteers consumed ¼c (phase 1, *n* = 24) or ½c (phase 2, *n* = 18) of black, cranberry, great northern, kidney, navy and pinto beans and corn, rice, pasta and potato (controls), with blood glucose measured before and for 2 h after eating. GRs (incremental areas under the curves, iAUCs) after beans were consumed were compared to those of controls by ANOVA followed by Dunnett’s test. To qualify for MED, beans had to elicit an effective reduction in GR, defined as a statistically significant reduction in iAUC of ≥20% (i.e., a relative glycemic response, RGR, ≤80). Outcomes from in vitro digestion were compared with in vivo RGR. Both doses of all six beans effectively reduced GR versus all four starchy controls, except for ¼c and ½c cranberry and pinto vs. corn, ¼c great northern and navy vs. corn and ¼c navy and pinto vs. potato. MED criteria were met for 18 comparisons of the ¼c servings, with four of the remaining six met by the ½c servings. The overall mean ± SEM RGR vs. controls was similar for the ¼c and ½c servings: 53 ± 4% and 56 ± 3%, respectively. By multiple regression analysis, RGR = 23.3 × RDS + 8.3 × SDS − 20.1 × RS + 39.5 × AS − 108.2 (rapidly digested starch, *p* < 0.001; slowly digested starch, *p* = 0.054; resistant starch, *p* = 0.18; available sugars, *p* = 0.005; model r = 0.98, *p* = 0.001). RGR correlated with in vitro glucose release (r = 0.92, *p* < 0.001). The MED of beans is ¼ cup. For *n* = 30 comparisons (*n* = 24 beans vs. controls, *n* = 6 controls vs. each other), an effective reduction in GR was predicted from in vitro carbohydrate analysis with 86% sensitivity and 100% specificity.

## 1. Introduction

*Phaseolus vulgaris* species, such as black, kidney, great northern, and pinto beans, are the dried seeds from leguminous crops that belong to the pulse family. They are low in fat and high in dietary fiber, protein, complex carbohydrates and micronutrients [1,2]. Regular consumption of diets that include beans is associated with a reduced risk of type 2 diabetes mellitus (T2DM), possibly by lowering glycemic response and improving glycemic control [3,4,5]. Beans are also recommended for dietary management of chronic disorders, such as obesity, cardiovascular disease and cancer [3,4]. Despite the diverse health-promoting attributes of beans and other pulses, they are consumed daily by only 13% of the Canadian adult population [6]. Pulse consumption could be promoted through public awareness of its associated health benefits, but this requires regulatory approval. The European regulatory authority has outlined the steps required to substantiate a claim for food products that lower postprandial blood glucose response (PBGR) [7,8]; however, gaps in the required evidence still exist. For example, it is important to demonstrate that a typical serving of a starchy food under consideration elicits a significantly lower blood glucose response compared to a similar carbohydrate-rich food [7,8].

Although beans are an important source of dietary protein, they are also an excellent source of complex carbohydrates that can be used to replace commonly consumed high-glycemic-index (GI) starchy foods to promote improved glycemic control [3,4,5]. Indeed, many studies have assessed the PBGR of beans with little regard for protein content. While these studies were standardized for available carbohydrate content, few compared the glycemic impact of a typical serving of beans to that of commonly consumed starchy foods [5,9,10,11]. The latter is needed to provide simple public-health-promotion messaging around beans and improved glycemic control. Additionally, there is a need for rigorous structure–function studies to elucidate the putative mechanisms by which beans and other pulses attenuate PBGR.

Beans contain several components that may influence the digestibility of their starch and potentially attenuate PBGR, which in turn could promote improved glycemic control [12,13,14,15]. In this regard, phenolic compounds have been shown to inhibit the starch digestive enzyme α-amylase [12] and to inhibit glucose absorption mechanisms [13]. Similarly, beans contain substantial amounts of slowly digested starch (SDS) and resistant starch (RS), which have been shown to slow the release and absorption of glucose [14,15], and total dietary fiber (TDF) that is resistant to enzymatic digestion. Indeed, a previous structure–function study showed that in vivo glycemic response after lentil consumption can be predicted from in vitro starch hydrolysis and a combination of rapidly digested starch (RDS) and RS contents [16].

The primary objective of this study was to determine the minimum effective dose (MED) of beans by comparing the glycemic responses elicited by ¼ cup or ½ cup servings of the six most commonly consumed cultivars of *Phaseolus* beans in Canada to those elicited by ¼ or ½ cup servings of four commonly consumed starchy control foods (corn, macaroni, instant white potato flakes and long-grain white rice), with the primary endpoint being the incremental area under the blood glucose response curve (iAUC) and the secondary endpoint being glucose peak rise. The secondary objective of this study was to determine whether in vivo iAUC could be predicted from the relative abundance of various carbohydrate fractions of the beans and starchy foods tested. We expect that this information would be useful in understanding the underlying mechanism by which beans lower PBGR, which is desirable for the approval of a health claim. It was hypothesized that the iAUC of beans would be significantly lower than that of starchy control foods and that in vivo responses would be inversely correlated with TDF and associated with in vitro digestion measures.

## 2. Materials and Methods

### 2.1. Test Foods

These were six Canadian beans: black turtle (black), cranberry (romano), great northern, navy (white), pinto and dark red kidney (kidney) beans provided by Ontario Bean Growers (Guelph, ON, Canada); four starchy control foods, including frozen corn (corn; Peaches and Cream Corn, President’s Choice, Loblaws Inc., Toronto, ON, Canada), macaroni (Elbows, Tradizionale, Italpasta Limited, Brampton, ON, Canada), instant white potato flakes (potato; Idahoan Original Mashed Potato, Idahoan Foods, Idaho Falls, ID, USA) and long-grain white rice (rice; Selection, Metro Brands, Montreal, QC, Canada).

### 2.2. Proximate Analysis

Raw foods (excluding corn, which was first freeze dried) were cracked, then ground using a Cyclone Sample Mill (UDY Corp., Fort Collins, CO, USA) equipped with a 1.0 mm screen and passed through a 250 µm sieve. Proximate analysis was performed in triplicate, except for potato, which was analyzed once, along with appropriate controls. With the exception of instant potato flakes, foods were analyzed at the Center of Excellence for Poultry Science Central Analytical Laboratory, University of Arkansas (Fayetteville, AR, USA) using standard Association of Official Agricultural Chemists (AOAC) official methods of analysis for ash (AOAC 923.03), protein (AOAC 990.03) and fat (AOAC 920.39). Energy was determined by a bomb calorimeter using method ANSI/ASTM D2015-77. Potato was analyzed at Maxxam Analytics International Corporation (Mississauga, ON, Canada) using standard methods for ash (AOAC 923.03), protein (AOAC 992.15) and fat (AOAC 922.06); carbohydrates and energy were mathematically derived. Moisture content of ground raw foods was determined using an Isotemp Vacuum Oven Model 280A (Fisher Scientific Company, Nepean, ON, Canada). For freshly cooked material, an HB43-S moisture analyzer was used (Mettler Toledo AG Laboratory & Weighing Technologies, Greifensee, Switzerland). Total starch (TS) was determined according to a modified version of AOAC Method 996. Cooked freeze-dried ground foods were used to measure dietary fiber, RS and free sugars (corn). Insoluble and soluble dietary fiber (IDF and SDF) were measured according to AOAC Method 2011.25. RS was measured according to AOAC method 2002.02, and mono- and disaccharides were extracted and analyzed as previously described [17]. Analytical kits for TS (K-TSTA), RS (K-RSTAR) and integrated TDF (K-GLUC) were purchased from Megazyme International Ireland Ltd., Co., Wicklow, Ireland.

### 2.3. In Vitro Digestion

In vitro digestion (IVD) was conducted using methods originally developed by Englyst [18] and Goni et al. [19] and modified for semi-automated analysis (Next Instruments, Condell Park. Australia) to directly measure the glucose released from freshly cooked foods using an on-board glucose analyzer (Analox Instruments Ltd., The Vale, London, UK). Cooked foods (see below) were minced using a CuisineArt Meat Grinder (Woodbridge, ON, Canada) through a 4.5 mm pore size disc, then equivalent volumes (0.0082 cup) were weighed into sample cups (120 mL) with 5 glass beads (4 mm diameter) and a magnetic stir bar. Samples were incubated at 37 °C with agitation (3000 rpm) throughout both the simulated gastric and intestinal phases of digestion using enzyme and reagent proportions as previously outlined [19] and included the incorporation of pepsin (180 mg) in the guar gum solution (4 mL) during the gastric phase. Cooked lentil powder was used as an internal quality control in each assay (200 mg). Samples were run in duplicate on 2 separate days to account for day-to-day variation.

### 2.4. In Vivo Glycemic and Insulin Response

Cooked foods used for human feeding trials were prepared using standardized weights and times as follows: (i) 90 g of each bean variety was weighed and soaked overnight at room temperature in distilled water (dH_2_O), drained, then cooked in a 6-cup rice cooker (Black & Decker Model RC3406C; The Black and Decker Corporation, Towson, MD, USA) in sufficient fresh dH_2_O to produce soft product with no excess liquid; (ii) 67.3 g of rice was rinsed, then directly cooked in a similar manner to the beans; (iii) 21 g of instant potato flakes was prepared using a weighed amount of boiling dH_2_O and stirred to a smooth consistency; (iv) macaroni and corn were prepared according to package directions.

Acute human feeding trials were conducted as previously outlined [20] to compare the glycemic response elicited by ¼ cup (42.7 to 46.1 g) or ½ cup (85.4 to 92.1 g) servings of 6 beans to those elicited by equivalent servings of 4 control foods: macaroni, corn, potato and rice (1/4 cup = 30.5 to 55.5 g and ½ cup = 61.7 to 115.1 g). This study was approved by the Western Institutional Review Board^®^ (Puyallup, WA, USA) and registered at clinicaltrials.gov as NCT02907190. Eligible participants provided written informed consent prior to starting the study. Eligible adults aged 18–75 years were recruited from Toronto (ON, Canada) and excluded if they had a history of AIDS, hepatitis, diabetes, a heart condition or food allergies.

The study was an open-label, randomized cross-over trial. All participants in both phases of the study tested each of the 6 beans in randomized order on visits 1, 3, 4, 6, 7 and 9, and 3 of the control foods, excluding corn, in randomized order on visits 2, 5 and 8. Since the difference in glycemic response between beans and the control foods was expected to be smallest for corn, all participants of both phases of the trial tested corn, while 16 of the 24 in the ¼ cup phase and 12 of the 18 in the ½ cup phase tested macaroni, potato and rice, so that each person tested 9 of the 10 different test meals. Participants were studied on 9 days, at least 2 days apart, over a period of 4 to 6 weeks. Portion sizes and composition of the ½ cup test meals are shown in Table 1.

Prior to consuming test meals, fasted (10 to 12 h) participants were weighed and 2 finger-prick blood samples were collected 5 min apart for glucose analysis. The meal was consumed within 10 min and was served along with a consistent drink of up to 1 cup of water or coffee or tea with 30 mL of 2% milk and non-caloric sweetener if desired. Following consumption, the participants remained seated quietly for the remainder of the 2 h test period. Additional blood samples were collected at 15, 30, 45, 60, 90 and 120 min following the first bite. Blood samples were also collected for insulin analysis at 0, 30, 60, 90 and 120 min from the group of 12 participants who consumed ½ cup of each bean and potato meal. Bloods collected for glucose analysis (2 to 3 drops into fluoro-oxalate tubes) were mixed, then stored at −20 °C until analysis within 3 days, using a YSI model 2300 STAT analyzer (Yellow Springs, OH, USA). Analytical coefficient of variation (CV) for blood glucose was calculated by measuring the 0 min sample twice. Bloods collected for insulin analysis (300 µL into Sarstedt Microvette 300) were left to clot at room temperature for at least 30 min, then centrifuged at 9000 rpm for 90 s; separated serum (approximately 100 µL) was transferred to polypropylene tubes and stored at −20 °C prior to analysis, according to the manufacturer’s instructions. Insulin ELISA kits (10-1113-10) and a 2-level antigen control set for human insulin (10-1164-01) were purchased from Mercodia AB (Uppsala, Sweden). Following consumption of the test meals, palatability was rated using a visual analogue scale.

### 2.5. Power Calculation

Study power was based on published carbohydrate content, estimated GI of beans and control food, and an assumption that beans would elicit a 22 to 26% lower glycemic response than corn and 38 to 48% lower glycemic responses than the other control foods. Assuming a within-individual CV of glycemic responses of 30% for ¼ cup servings and of 25% for the ½ cup servings, *n* = 24 or *n* = 18 participants, respectively, provided 80% power to detect a difference in glycemic response of 25% to detect the difference for corn, and *n* = 16 or *n* = 12 participants, respectively, provided 90% power to detect the difference in glycemic response of 37% for the other 3 control foods.

### 2.6. Statistical Analysis

SigmaPlot 14.0 (SYSTAT Software Inc, Delaware, USA) was used for data capture and analysis. Available carbohydrates (ACs) were calculated as the sum of (glucose, sucrose, fructose and galactose) plus (TS − RS) plus (glucose and ½ sucrose). IVD outcomes including RDS, SDS and RS were normalized to TS content (%, dwb). Area under the starch hydrolysis curve (SHAUC, mg.min (dwb)) and area under the glucose release curve (GHAUC, mg.min (fwb)) were calculated as previously described [16,19]. Mean differences among the in vitro variables were assessed by one-way ANOVA followed by Tukey’s multiple comparisons test if indicated.

Correlations between in vitro and in vivo variables for the ½ cup portions of beans (*n* = 18) and the controls (*n* = 18 for corn and *n* = 12 for macaroni, potato and rice) were assessed by univariate and multivariate regression analysis (Excel 365, Microsoft Corp., Redmond, WA, USA). The number of participants testing the control foods differed; the mean iAUC for corn in all 18 participants was 54.6 mmol×min/L, while the mean iAUCs for corn in the 12 participants who tested macaroni, potato and rice were 57.8, 55.3 and 50.8 mmol×min/L, respectively. To correct for this, the mean iAUCs for macaroni, potato and rice were normalized as follows: C18×F12/CF12, where C18 = mean iAUC for corn in 18 participants, F12 = mean iAUC for the food in 12 participants (i.e., 103.2, 67.4 and 107.0 mmol×min/L for macaroni, potato or rice, respectively and CF12 = the mean iAUC for corn in the 12 who tested the food (macaroni, potato or rice). Thus, the adjusted mean for macaroni was 54.6 × 103.2/57.7 = 97.5 mmol × min/L.

For the human trial, glucose and insulin iAUCs, ignoring the area below fasting, were calculated using the trapezoid rule [20]. Peak rise was defined as the maximum concentration of glucose measured during the 2 h test minus the fasting concentration. Fasting glucose was taken to be the mean of the concentrations in the 2 fasting samples. Of the 1744 planned blood samples, 12 samples could not be analyzed due to clots; 2 missing samples at −5 min were replaced by the 0 min blood glucose value; 9 missing values from 15 and 90 min were replaced with the mean of the surrounding blood glucose values, and for the 1 missing value at 120 min, the 90 min blood glucose value was carried forward. The intra-assay CV for glucose using −5 and 0 min samples was 3.1% (*n* = 214). For insulin analysis, the intra-assay CV (*n* = 14) was 4.5% and the inter-assay CV (*n* = 7) was 3.0%.

Treatment effects of glycemic responses in the ¼ cup phase were compared using 4 separate sets of data; 1 set consisted of the *n* = 24 participants who consumed corn and all 6 beans, and the other 3 sets consisted of the *n* = 18 participants who consumed macaroni and all 6 beans, potato and all 6 beans, or rice and all 6 beans. Similarly, the 4 data sets in the ½ cup phase consisted of the 18 participants who tested corn and the 12 participants who tested macaroni, potato or rice. Data were assessed for normality using the observed mean and SD by comparing the distribution of values in bins 1 × SD wide from <2.5 × SD below the mean to >2.5 × SD above the mean with the theoretical normal distribution using the chi-squared test. Values not normally distributed were normalized by square-root transformation prior to statistical testing. Data were subjected to repeated-measures ANOVA examining for the main effect of test meal. After a demonstration of significant heterogeneity, the means for the 6 beans were compared to their respective controls using Dunnett’s test to adjust for multiple comparisons (Prism, GraphPad Software, La Jolla, CA, USA). The *p*-values from Dunnett’s test were multiplied by 4 (Bonferroni’s correction) to adjust for the fact that the data from each phase were subjected to 4 separate ANOVAs. The criterion for significance was a Bonferroni-adjusted 2-tailed *p* < 0.05. Results for normally distributed data are reported as means ± SEMs; results for square-root-transformed data are reported as medians (interquartile ranges). Relative glycemic response (RGR) was calculated as the mean (or median) iAUC of each bean expressed as a percentage of that for its respective control food. The MED of a bean was defined as the lowest dose that elicited an effective reduction in GR, defined as a statistically significant reduction in iAUC of ≥20% relative to its control (i.e., an RGR ≤ 80%).

## 3. Results

### 3.1. Nutrient Composition

Table 1 shows the nutrient composition of the test meals as eaten for the ½ cup phase of the human trial. Overall, the beans contained significantly more protein, energy and TDF than the control foods (*p* < 0.05). The carbohydrate contents of beans, rice and macaroni were similar but significantly higher (*p* < 0.05) than those of corn and potato, while the ACs of beans, corn and potato were significantly less (*p* < 0.05) than those of rice and macaroni. Dietary fiber fractions, along with TS, available sugar (AS) and RS contents per 100 g dry weight of foods, are summarized in Table 2.

### 3.2. In Vitro Glucose and Starch Digestibility

Table 2 shows the results for the in vitro digestion of the beans and control foods. Analytical variation as assessed with an internal quality-control food sample (*n* = 16) for RDS, SDS and RS fractions was 4.1%, 14.2% and 11.6%, respectively. The control foods were digested faster than the beans, with instant potato being the fastest; within the first 20 min, approximately 90% of the starch was digested (i.e., RDS). Among the beans, pinto had the highest rate of digestion, which was comparable to rice and corn (*p* < 0.05). After 2 h of digestion, approximately 80 to 90% of the starch in each food was converted to glucose. Among the beans, black beans contained the least RDS and pinto the most. The RDS of control foods was significantly higher (*p* < 0.05) than that of beans, with the exception of pinto, which was similar to corn, macaroni and rice. The SDS contents of the beans averaged 40%, with black beans containing the most SDS and pinto the least. The SDS of control foods was significantly higher (*p* < 0.05) than that of beans, while potato contained the least. The RS values of foods, as measured by IVD methods, were higher than those measured directly. The GHAUCs and SHAUCs resulting from in vitro digestion of beans and control foods at equivalent serving sizes (0.0082 cup) are shown in Table 2. As expected, the SHAUCs of beans, except pinto, were significantly less (*p* < 0.05) than those of the control foods. Similarly, the GRAUCs of beans, except for cranberry, were significantly lower (*p* < 0.05) than those of the starchy controls.

### 3.3. In Vivo Glycemic Responses

#### 3.3.1. The ¼ Cup Servings

**Figure 1:**
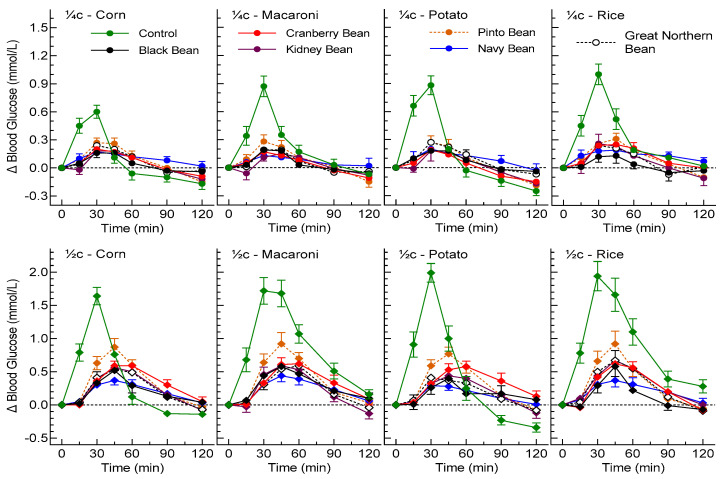
Glycemic responses elicited by ¼ and ½ cup servings of beans and controls. Values are means ± SEMs for *n* = 24 (¼c corn), *n* = 16 (¼c macaroni, potato and rice), *n* = 18 (½c corn) or *n* = 12 (½c macaroni, potato and rice).

Twenty-four participants finished the ¼ cup phase of the study; individual details are shown in Table 3. Among the participants, two used supplements (fish oil; probiotics, vitamin D and fish oil) and four were on allowed prescription medications. There was no significant difference in fasting glucose among treatments, varying from a mean of 4.41 to 4.55 mmol/L. The incremental glycemic responses after consumption of ¼ cup servings of the beans compared to equivalent servings of the control foods are shown in the top four panels of Figure 1. Peak blood glucose occurred at 30 min for all foods and was not different among the beans. However, peak glucose levels after corn, rice, macaroni and potato were significantly greater (*p* < 0.001) than those after beans (Table 4).

The distribution of the iAUC values for the ¼ cup servings was not normal but was normalized by square-root transformation. The median iAUCs elicited by the beans compared to the control foods for the ¼ cup serving size are given in Table 5. Median iAUCs after black and kidney beans were significantly less than that after corn; median iAUCs after black, cranberry, great northern and kidney beans were significantly less than that after potato, and median iAUCs after all six beans were significantly less than those after macaroni and rice. The RGRs of all beans versus their respective controls were ≤80% (range: 25 to 80%) for all comparisons, except for pinto beans (RGR = 85%) vs. corn. By Bonferroni-adjusted paired *t*-test, the median iAUC of all six beans was less than that of their respective controls (*p* < 0.01), with the mean RGR of all six beans being 70% versus corn (*p* = 0.006), 42% versus macaroni (*p* < 0.001), 45% versus potato (*p* < 0.001) and 37% versus rice (*p* < 0.001).

#### 3.3.2. The ½ Cup Servings

The participants’ characteristics are shown in Table 3. There was no significant difference in fasting glucose among the treatment groups, with the means varying from 4.41 to 4.55 mmol/L. The bottom four panels of Figure 1 show the incremental glycemic responses following consumption of ½ cup servings of the beans or control foods. Glucose peak rises following ½ cup servings (Table 4) for all six beans were significantly lower (*p* < 0.001) those of the control foods. Table 5 shows the results for glucose iAUC and RGR for all beans and controls foods and for insulin iAUC and relative insulin responses (RIRs) for the potato study.

The iAUC values after the ½ cup servings did not differ significantly from a normal distribution. As shown in Table 5, ½ cup servings of all six beans elicited significantly lower glycemic responses than all four of their respective controls, except for cranberry and pinto beans vs. corn. The RGRs of all beans versus their respective controls were ≤80% (range: 25 to 80%) for all comparisons. By Bonferroni-adjusted paired *t*-test, the median iAUC for all six beans was less than that for their respective controls (*p* < 0.01), with the mean RGR of all six beans being 62% versus corn (*p* = 0.002), 36% versus macaroni (*p* < 0.001), 45% versus potato (*p* < 0.001) and 32% versus rice (*p* < 0.001).

The mean ± SEM RGR for the *n* = 6 × ½ c servings did not differ significantly from the *n* = 6 × ¼ c servings, respectively, for beans vs. corn, 62 ± 5% vs. 70 ± 5%; beans vs. macaroni, 36 ± 3% vs. 39 ± 3%; beans vs. potato, 45 ± 5% vs. 44 ± 5%; and beans vs. rice, 32 ± 3% vs. 36 ± 2%.

### 3.4. Insulin Responses

**Figure 2:**
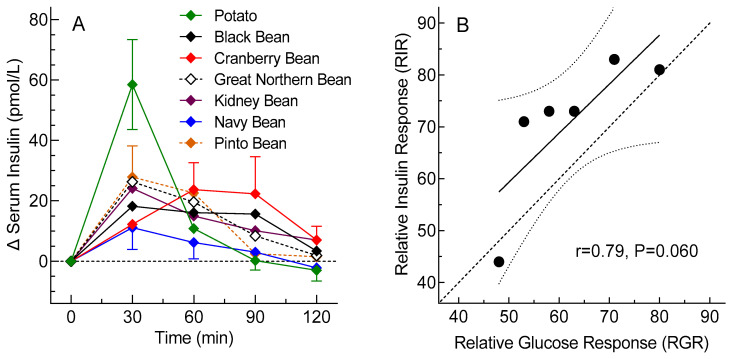
Serum insulin responses for ½ cup servings of beans versus potato. (**A**) Incremental serum insulin responses, means ± SEMs for *n* = 12 participants. (**B**) Relationship between relative insulin responses and relative glucose responses for the ½ cup servings of six beans relative to ½ cup of potato. The dashed line is the line of identity, the solid line is the regression line and the dotted lines are the 95% confidence intervals of the regression line.

Since the glycemic response for potato was expected to be the highest among the control foods, insulin was measured only for the potato study. Mean fasting insulin across the seven treatments varied from 20 to 26 pmol/L (*p* = 0.57). Postprandial insulin increments are shown in Figure 2A. The insulin iAUC values did not differ significantly from a normal distribution and their means did not differ significantly among treatments (*p* = 0.30; Table 5). The distribution of insulin peak rises was normalized by square-root transformation, and the transformed means differed significantly among treatments (*p* = 0.006), with those for black and navy beans being significantly less than that for potato (Table 4). The mean ± SEM RIR of the six beans relative to potato, 71 ± 14%, did not differ significantly from the RGR for the six beans relative to potato, 62 ± 12% (*p* = 0.31). Although the correlation between RIR and RGR was not significant (*p* = 0.06), the regression line did not differ significantly from the line of identity, with the mean (95% confidence interval) slope, 0.94 (−0.05, 1.94), not differing significantly from 1 and the y-intercept, 12 (−51, 75), not differing significantly from 0 (Figure 2B).

### 3.5. Palatability

In both the ¼ cup and ½ cup phases of the trial, corn was rated as being significantly more palatable than beans (*p* < 0.05). However, beans were rated as being of equal or higher palatability than the other control foods, although the differences were not statistically significant.

### 3.6. Relationships between Food Components and between In Vitro and In Vivo Variables

**Figure 3:**
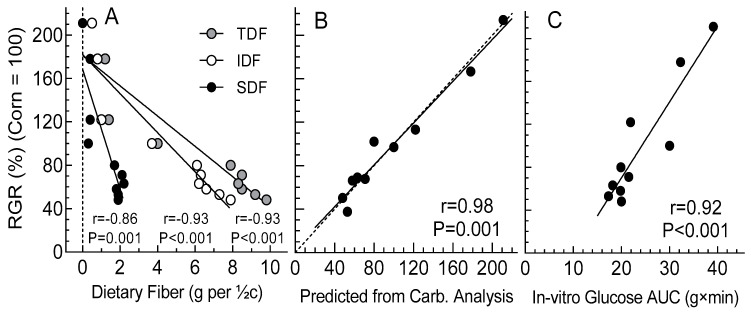
Relationships between glycemic response and in vitro analysis of beans and controls. Correlation coefficients (*n* = 10) and regressions for mean RGRs (relative glycemic responses) elicited by the ½ cup servings of the beans and control foods relative to corn: *n* = 18 for corn and the six beans, and *n* = 12 (drawn from the pool of *n* = 18) for macaroni, potato and rice. The mean iAUCs for macaroni, potato and rice were adjusted to account for the different numbers of participants in which they were tested, as described in the Methods section, and the RGR relative to corn was calculated. (**A**) shows the relationships between RGR and insoluble (IDF), soluble (SDF) and total dietary fiber (TDF). (**B**) shows the relationship between the observed RGR and that predicted by multiple regression analysis from the amounts of rapidly digested starch (RDS), slowly digested starch (SDS), resistant starch (RS) and available sugars (AS) contained in the ½ cup test means as follows (SE of the y estimate = 14.6): RGR = 23.3 × RDS + 8.3 × SDS − 20.1 × RS + 39.5 × AS − 108.2. The dashed and solid lines are the line of identity and the regression line, respectively. (**C**) shows the relationship between RGR and the area under the curve of glucose released during in vitro digestion (GHAUC) of the beans and control foods.

Correlations between in vitro digestion and in vivo blood glucose response were similar for ¼ cup and ½ cup servings; as such, only results for ½ cup servings are described. The ½ cup servings of corn, macaroni, potato and rice contained an average of 8.0, 17.3, 10.9 and 21.1 g avCHO compared to 9.0 to 12.0 g for the ½ cup servings of beans (Table 1). There was a significant relationship between the amount of avCHO consumed and RGR (r = 0.862, *p* = 0.001, *n* = 10, not shown). Mean RGR was inversely related to SDF (r= −0.86, *p* =0.001, *n* = 10), IDF (r = −0.93, *p* < 0.001, *n* = 10) and TDF (r = −0.93, *p* < 0.001, *n* = 10; Figure 3A) by multiple regression analysis. Figure 3B shows that observed RGR could be predicted with a high degree of certainty (r = 0.98; p = 0.001) using an regression equation derived from RDS, SDS, RS and available sugars (AS). Further, as shown in Figure 3C, there was a significant relationship between RGR and the area under the curve of glucose released during in vitro digestion (GHAUC) of the beans and control foods.

### 3.7. Minimum Effective Dose

The MED for beans to elicit an effective glucose reduction (eGR) versus corn was achieved with ¼ c servings of black and kidney beans and with ½ c servings of great northern and navy beans, but was not achieved with either dose of cranberry or pinto beans (Table 5). The MED for beans to elicit an eGR versus both macaroni and rice was achieved with ¼ c servings of all six beans tested (Table 5). The MED for beans to elicit an eGR versus potato was achieved with ¼ c servings of black, cranberry, great northern and kidney beans and with ½ c servings of navy and pinto beans (Table 5). Thus, the MED criteria (statistically significant reduction in glucose iAUC of ≥20%) were met for 22 of the 24 comparisons of beans vs. controls, with 18 of these (82%) achieved by the ¼c dose. 

### 3.8. Prediction of eGR from Carbohydrate Analysis

We defined an eGR as a significant reduction in iAUC of ≥20% (i.e., an RGR ≤ 80%). To determine whether carbohydrate analysis could predict the existence of an eGR, in vitro RGRs were calculated as ivRGR = SE + 100 ∗ Fe/Ce, where SE is the standard error of the y estimate from the multiple regression analysis (14.6) and Fe and Ce were the y estimates from the multiple regression equation (footnote to Figure 3B) for a food and its comparator, respectively. The observed RGRs for ½ cup beans vs. controls are given in Table 5. The post hoc observed RGRs among the ½ cup controls (paired *t*-test) were: corn vs. macaroni, potato and rice (*n* = 12), 56% (*p* < 0.001), 82% (ns) and 48% (*p* < 0.001), respectively; macaroni vs. potato and rice (*n* = 6), 74% (ns) and 99% (ns), respectively; and potato vs. rice (*n* = 6), 72% (*p* = 0.01). The observed differences in GR for 25 of the 30 total comparisons constituted an eGR. Based on the ivRGRs, 22 predictions were true positives (correct prediction of an eGR), 5 were true negatives (correct prediction of no eGR) and 3 were false negatives (incorrect prediction of no eGR). None of the 24 in vitro estimates were false positives (incorrect prediction of an eGR) (Table 6). Thus, the sensitivity of the ivRGR (number of true-positive predictions, *n* = 22, as a percentage of the number of actual positive results, *n* = 25) was 86% and its specificity (number of true negative predictions, *n* = 5, as a percentage of the number of actual negative results, *n* = 5) was 100%. Although these estimates are not very precise due to the small number of tests on which they are based (*n* = 30), they suggest that the in vitro method described has reasonably good sensitivity and specificity to predict the existence of an eGR.

## 4. Discussion

This study sought to determine the MED of six beans required to significantly lower glycemic response compared to four commonly consumed starchy foods, as well as to improve our understanding of the influence of bean carbohydrate fractions on blood glucose. MED was defined as the smallest dose that elicited a statistically significant reduction in mean glucose iAUC of at least 20% relative to the control food. The results showed that the MED criteria were met with ¼ cup servings of beans for 18 (75%) of the 24 possible comparisons of beans vs. controls, with the MED criteria being met with ½ cup servings of beans for four of the remaining six comparisons. The only comparisons not meeting the MED criteria were those for cranberry and pinto beans compared to corn.

The novel inclusion of corn as a control food in an acute human feeding trial, as opposed to potato, rice and pasta, which have been often used [5,21,22], is important since it has a carbohydrate profile similar to beans and is often consumed as a side dish. However, the corn test meal contained less avCHO than macaroni and rice and had a lower GI than instant potato [9]; this partly explains why the underlying difference between corn and beans was smaller than that between the beans and the other control foods. It is also possible that the corn variety used in this study is less digestible due to a highly branched glycogen-like structure of starch [23], and although it would be useful to assess other varieties, corn provides an objective comparison for evaluating the PBGR-lowering effect of beans. The insulin response elicited by the various beans tended to be less than that for potato, with the insulin peak rise being significantly lower after black and navy beans compared to potato (Table 4). This is similar to the finding of Winham et al. [24] in a human study that assessed insulin response following consumption of rice or beans. The lack of significant differences between the mean RIR and RGR values for the six beans and the positive correlation between RIR and RGR (Figure 2B) provides no good evidence for a disproportionately increased insulin response. Thus, the reduced PBGR elicited by beans is unlikely to be the result of an increased secretion of insulin and is attributable to an intrinsic property of the beans tested [25].

The PBGR-attenuating effect of navy beans was previously established in healthy participants following consumption of various pulses when compared to a pasta and tomato sauce control and standardized for energy content but not serving size [21]. Similarly, the PBGR of navy bean powder combined with tomato sauce was also found to be significantly lower than that of a wholewheat flour control [22]; the study controlled for AC but serving sizes were different. The latter is an important consideration in creating regulatory guidelines for a health claim about bean consumption and PBGR lowering [7,8] and for providing population simplified messaging to promote bean consumption [26].

Our study also sought to determine the minimum effective dose of beans required to significantly lower PBGR. In addition to demonstrating significant dose–response differences in iAUC, it was important to provide a simple measure of the magnitude by which beans reduced iAUC in relation to the control foods. We used RGR, which is analogous to GI, to reflect the extent to which the iAUC of beans was lower than that of each control food. We reasoned that, if a serving of beans elicits a lower glycemic response than a serving of another food, then any fraction of the serving of the same beans will elicit a lower response than the same fraction of a serving of the same other food. However, as serving size decreases, the absolute difference in glycemic response also decreases, and the resulting smaller signal-to-noise ratio of the measured glycemic response lowers the statistical power to detect the difference [27]. Additionally, the expected difference in glycemic response is dependent on the avCHO content in the servings of beans and the comparison food and their GI values. The smaller the expected difference in glycemic response, the less statistical power there will be to detect the difference, and therefore either larger portion sizes will be required (yielding a larger absolute difference and lower variation) or a larger number of participants. The validity of these considerations has been demonstrated in this study.

In choosing a ¼ cup serving as the lower serving of beans, we inspected the relevant literature and found that most human trials that examined the PBGR lowering of beans used 90 and 465 g to assess this effect [6]. Our study shows that ½ cup of beans weighs about 97 g, on average, which is similar to that reported by Doma et al. [28], but very few studies have studied the PBGR of less than ½ cup of beans. Due to the reduced power to detect a small difference for the ¼ cup servings, none of the individual comparisons between beans and corn was significantly different. However, the greater power to detect the larger absolute difference with the ½ cup servings resulted in most of those differences being statistically significant, even though the percent difference tended to be smaller for the ½ cup compared to the ¼ cup servings. Thus, we could conclude that the minimum effective dose of beans to lower the glycemic response compared to corn is ≥½ cup. However, since the differences between beans and macaroni, corn and potato were larger than that between beans and corn, there was enough power in the ¼ cup series to demonstrate statistically significant differences. Thus, the minimum effective dose of beans to lower the glycemic response compared to macaroni, rice or potato is ≤¼ cup.

It is important to consider the magnitude of the reduction in PBGR by beans in terms of statistical and clinical significance. The mean RGR of ½ cup servings of beans varied from 32% vs. rice to 62% vs. corn, indicating that beans reduced iAUC by 38 to 68% compared to the controls—differences that were statistically significant. Further, the mean RGRs of ¼ cup servings of beans were 37–70%, indicating that iAUC was reduced by 30 to 63% compared to the control starchy foods. At an individual level, compared to corn, ½ cup of cranberry beans had a 29% lower RGR, while that of pinto beans was 20% less, but these values were not significantly different. Regardless of the level of significance, these treatment effects lower glycemic response, and if beans are used to replace some of these starchy controls, it is expected that clinically significant improvements in glycemic control will accrue [3,4,5].

Overall, the results from the nutrient composition analysis and the in vitro starch hydrolysis of test foods are consistent with previous work [29,30,31,32]. However, some discrepancies were observed between amounts of starches and dietary fiber, possibly due to differences in sample preparation techniques as well as analytical methods. The original protocol of Englyst et al. [18] lacks a gastric incubation phase with pepsin and HCl solution and may have resulted in the incomplete digestion of the bean starches, causing underestimated values of RDS and overestimated values of SDS, as were observed by others [31,32]. The effect of the method of analysis on measurements of starch fractions was further evident from the slight differences we observed between the results of the two methods used to measure the RS content (derived via IVD vs. direct via AOAC). The nutrient analysis and in vitro starch hydrolysis of six beans and four control foods showed significant differences in protein, dietary fiber, TS and starch fractions (RDS, SDS and RS). These differences were used to further investigate the predictors of human blood glucose responses. Previous evidence suggests that dietary fiber, especially TDF, may be used as a strong predictor of glycemic response [33]. In support of these findings, our analysis showed strong negative correlations between blood glucose response and IDF, SDF and TDF.

Our results indicate that in vivo glucose and insulin response were significantly related to in vitro digestion. Nevertheless, since the regression of GHAUC did not go through the origin, it may provide a qualitative but not quantitative predictor of in vivo blood glucose response. Relationships between RDS and blood glucose response and GI have been observed previously, which suggests the possibility of its use as a predictor [34,35]. However, we found that by including in vitro measurements of RDS, SDS, RS and AS in a multiple regression analysis, the RGRs of the 10 foods tested could be predicted accurately enough to allow prediction, with a relatively high degree of sensitivity and specificity, of whether a ½ cup serving of beans would elicit a significant ≥20% lower glycemic response than a ½ cup serving of a starchy control food. In showing significant reduction in glucose response by beans compared to control foods, these results from our in vitro and in vivo analyses add to the evidence for the blood-glucose-lowering effect of beans. It is important to continue to investigate the underlying mechanism by which this effect is achieved [16].

## 5. Conclusions

This study examined the minimum effective dose of the six most commonly consumed beans required to significantly attenuate PBGR compared to commonly consumed starchy foods. The minimum effective dose of beans to lower the glycemic response compared to corn is ≥½ cup. However, since the differences between beans and macaroni, corn and potato were larger than that between beans and corn, there was enough power in the ¼ cup studies to demonstrate statistically significant differences. Thus, the minimum effective dose of beans to lower the glycemic response compared to macaroni, rice or potato is ≤¼ cup. It is important for industry and health promoters to use this information in order to make accurate claims and guide consumers towards healthy food choices.

The beans contained higher amounts of dietary fiber, lower amounts of RDS, and higher levels of SDS and RS than the control foods, resulting in lower glycemic responses. Correlation between in vivo and in vitro responses showed that dietary fiber and GHAUC can be used as qualitative predictors of human glycemic response and that RDS, SDS, RS and AS may provide moderately accurate quantitative prediction.

## Figures and Tables

**Table 1:** Nutrient composition of test meals as eaten, ½ cup servings ^1^.

Test Meal	Serving, g	MC, g	Protein, g	Ash, g	Fat, g	Energy, kJ	CHO, g	AC, g	TDF, g
Beans ^2^									
Black	89.2	53.9 ± 0.2	7.6 ± 0.0 ^b^	1.81 ± 0.01 ^a^	0.49 ± 0.03 ^cd^	655 ± 1 ^b^	25.4 ± 0.1 ^b^	10.0 ± 0.1 ^ef^	9.2 ± 0.2 ^b^
Cranberry	92.0	58.8 ± 0.1	6.6 ± 0.0 ^e^	1.64 ± 0.01 ^c^	0.44 ± 0.01 ^e^	608 ± 1 ^c^	24.4 ± 0.0 ^c^	10.1 ± 0.1 ^e^	8.5 ± 0.1 ^c^
Great northern	92.1	58.6 ± 0.1	8.3 ± 0.0 ^a^	1.80 ± 0.00 ^a^	0.45 ± 0.01 ^de^	614 ± 1 ^c^	23.0 ± 0.0 ^f^	9.7 ± 0.4 ^f^	8.5 ± 0.2 ^c^
Kidney	85.4	52.7 ± 0.2	7.3 ± 0.0 ^c^	1.62 ± 0.00 ^c^	0.43 ± 0.02 ^e^	599 ± 1 ^d^	23.2 ± 0.0 ^e^	9.0 ± 0.4 ^g^	8.3 ± 0.4 ^cd^
Navy	90.4	54.0 ± 0.3	7.2 ± 0.0 ^d^	1.79 ± 0.02 ^a^	0.67 ± 0.03 ^b^	673 ± 5 ^a^	26.8 ± 0.0 ^a^	12.0 ± 0.0 ^c^	9.8 ± 0.1 ^a^
Pinto	90.7	59.2 ± 0.1	6.7 ± 0.1 ^e^	1.68 ± 0.01 ^b^	0.51 ± 0.02 ^c^	575 ± 1 ^e^	22.6 ± 0.0 ^g^	10.5 ± 0.3 ^de^	7.9 ± 0.1 ^d^
Controls									
Corn	79.9	58.2 ± 0.3	2.9 ± 0.0 ^g^	0.75 ± 0.01 ^d^	2.33 ± 0.02 ^a^	438 ± 1 ^h^	15.7 ± 0.0 ^i^	8.0 ± 0.1 ^h^	4.0 ± 0.2 ^e^
Macaroni	61.0	36.6 ± 0.2	3.5 ± 0.0 ^f^	0.23 ± 0.00 ^f^	0.07 ± 0.01 ^f^	452 ± 1 ^g^	20.7 ± 0.0 ^h^	17.3 ± 0.2 ^b^	1.2 ± 0.1 ^f^
Potato	115.1	99.5 ± 0.1	1.6 ^h^	0.58 ^e^	0.06 ^f^	247.0 ^i^	13.4 ^j^	10.9 ± 0.0 ^d^	1.4 ± 0.1 ^f^
Rice	87.1	61.6 ± 1.0	1.1 ± 0.0 ^i^	0.12 ± 0.01 ^g^	0.05 ± 0.01 ^f^	458 ± 1 ^f^	24.2 ± 0.0 ^d^	21.1 ± 0.0 ^a^	0.5 ± 0.0 ^g^

^1^ Values are means ± SDs (*n* = 3), except for potato (*n* = 1). ^abcdefghi^ Means in the same column with different superscripts differ significantly (*p* < 0.05, Tukey’s test). ^2^ Beans were soaked for 17 to 18 h at room temperature prior to cooking. Proximate analysis completed by the Central Analytical Laboratory, University of Arkansas, Fayetteville, AR, USA, expect for potato, which was analyzed by Maxxam Analytics International Corporation, Mississauga, ON, Canada. Methods used: protein, AOAC 992.15; ash, AOAC 923.03; fat, AOAC 922.06; CHO, by difference; energy (excluding potato), AOAC ANSI/ASTM D2015-77, potato, calculation. AC analysis was in house and is a summation of total starch (AOAC 996.11) minus resistant starch (AOAC 2002.02) plus glucose and ½ sucrose [17]; glucose and sucrose contents of macaroni obtained from Canadian Nutrient File (Food Code: 4506). AC, available carbohydrate; CHO, carbohydrate; MC, moisture content; TDF, total dietary fiber.

**Table 2:** Dietary fiber, total starch, available sugars, resistant starch and in vitro digestion outcomes of beans and control foods ^1^.

Sample	Direct Assay (dwb)						In Vitro Digestion Assay (dswb)				As Eaten (Fresh Weight)
	IDF, %	SDF, %	TDF, %	TS, %	AS, %	RS, %	RDS, %	SDS, %	RS, %	SHAUC, mg.min	GRAUC, mg.min
	(*n* = 3)	(*n* = 3)	(*n* = 3)	(*n* = 3)	(*n* = 3)	(*n* = 3)	(*n* = 4)	(*n* = 4)	(*n* = 4)	(*n* = 4)	(*n* = 4)
Beans											
Black	20.8 ± 0.5 ^ab^	5.3 ± 0.1 ^c^	26.1 ± 0.6 ^ab^	38.9 ± 0.5 ^de^	1.0 ± 0.0 ^d^	11.5 ± 0.5 ^ab^	34.8 ± 1.7 ^f^	50.8 ± 3.7 ^a^	14.8 ± 3.5 ^abc^	7451 ± 526 ^f^	17,293 ± 1049 ^d^
Cranberry	19.0 ± 0.4 ^c^	6.4 ± 0.6 ^ab^	25.5 ± 0.2 ^ab^	40.4 ± 0.5 ^cd^	1.5 ± 0.1 ^b^	11.3 ± 0.2 ^bc^	47.5 ± 0.5 ^e^	38.9 ± 1.2 ^bc^	13.7 ± 1.0 ^abc^	8456 ± 420 ^de^	21,532 ± 806 ^c^
Great northern	19.8 ± 03 ^bc^	5.4 ± 0.5 ^bc^	25.2 ± 0.7 ^b^	39.7 ±0.8 ^cd^	1.4 ± 0.1 ^bc^	12.1 ± 0.5 ^a^	54.7 ± 2.5 ^d^	28.1 ± 2.5 ^d^	17.2 ± 0.2 ^a^	8347 ± 129 ^e^	19,818 ± 310 ^cd^
Kidney	19.0 ± 1.2 ^c^	6.6 ± 0.3 ^a^	25.5 ± 1.2 ^ab^	36.8 ± 1.4 ^e^	1.4 ± 0.0 ^bc^	10.6 ± 0.3 ^c^	54.4 ± 4.1 ^de^	34.2 ± 3.0 ^bc^	11.3 ± 2.1 ^abc^	8710 ± 317 ^cde^	18,216 ± 1000 ^d^
Navy	21.7 ± 0.6 ^a^	5.3 ± 0.3 ^c^	26.9 ± 0.3 ^a^	41.5 ± 0.6 ^c^	1.3 ± 0.0 ^c^	9.9 ± 0.3 ^d^	39.5 ± 4.5 ^f^	42.3 ± 6.6 ^ab^	18.2 ± 5.6 ^a^	7435 ± 446 ^f^	20,048 ± 1572 ^cd^
Pinto	19.5 ± 0.1 ^bc^	5.5 ± 0.6 ^bc^	25.0 ± 0.5 ^b^	41.7 ±0.6 ^c^	1.5 ±0.1 ^b^	9.7 ± 0.7 ^d^	62.2 ± 1.6 ^bc^	27.3 ± 1.5 ^de^	10.6 ± 0.6 ^abc^	9197 ± 89 ^bcd^	19,871 ± 1876 ^cd^
Controls											
Corn	17.1 ± 0.7 ^d^	1.2 ± 0.1 ^e^	18.3 ± 0.8 ^c^	25.9 ± 0.3 ^f^	15.1 ± 0.6 ^a^	4.1 ± 0.2 ^e^	64.8 ± 1.4 ^bc^	19.1 ± 4.6 ^e^	16.1 ± 4.4 ^ab^	8825 ± 328 ^bcde^	29,967 ± 723 ^b^
Macaroni	3.3 ± 0.1 ^f^	1.6 ± 0.1 ^de^	4.9 ± 0.2 ^e^	72.1 ± 2.8 ^b^	0.5 ^f^	1.9 ± 0.1 ^f^	68.7 ± 3.2 ^b^	19.8 ± 3.0 ^e^	11.5 ± 2.5 ^abc^	9470 ± 261 ^b^	32,309 ± 1995 ^b^
Potato	6.5 ± 0.2 ^e^	2.5 ±0.3 ^d^	9.0 ± 0.4 ^d^	71.9 ± 1.3 ^b^	0.7 ± 0.1 ^e^	2.5 ± 0.1 ^f^	89.1 ± 1.7 ^a^	2.9 ± 1.9 ^f^	8.0 ± 3.3 ^bc^	10,527 ± 265 ^a^	21,894 ± 817 ^c^
Rice	1.9 ± 0.1 ^f^	0.1 ± 0.0 ^f^	2.0 ± 0.1 ^f^	83.5 ± 2.2 ^a^	0.1 ± 0.0 ^g^	0.9 ± 0.0 ^g^	60.7 ± 2.2 ^cd^	31.3 ± 2.0 ^cd^	8.0 ± 2.9 ^c^	9277 ± 310 ^bc^	39,052 ± 1756 ^a^

^1^ Values are means ± SDs (*n* = 3 or 4). ^abcdefg^ Means in the same column with different letter superscripts differ significantly (*p* < 0.05 Tukey’s test). Dietary fiber, RS (direct assay) and AS (of corn) were determined for samples that were cooked, freeze-dried, then ground (≤600 µm particle size). TS and AS (excluding corn) were determined for raw ground samples. Starch fractions (RDS, SDS and RS) were determined for equivalent volumes (0.0082 cup) of fresh cooked foods. AS, available sugar; dswb, dry starch weight basis; dwb, dry weight basis; GRAUC, in vitro glucose release area under the curve; IDF, insoluble dietary fiber; RS, resistant starch; RDS, rapidly digestible starch; SDF, soluble dietary fiber; SDS, slowly digestible starch; SHAUC, starch hydrolysis area under the curve; TDF, total dietary fiber; TS, total starch.

**Table 3:** Participant details: ½ and ¼ cup phases of study.

Sex	Ethnicity	Age,	Height,	Weight,	BMI,
		yrs	cm	kg	kg/m^2^
½ cup phase					
F	Caucasian	48	155.5	67.6	28.0
M	Chinese	54	170.0	78.9	27.3
M	Caucasian	25	181.5	81.3	24.7
M	Caucasian	25	183.5	79.2	23.5
M	Caucasian	57	176.6	79.0	25.3
M	Caucasian	56	170.7	78.4	26.9
F	Black	47	158.5	56.0	22.3
M	Black	28	174.0	74.5	24.6
F	Black	49	170.4	73.5	25.3
F	Black	37	162.0	66.9	25.5
M	Chinese	19	166.0	86.2	31.3
F	South East Asian	36	159.5	59.8	23.5
F	Caucasian	27	166.0	64.2	23.3
M	Caucasian	25	189.5	83.5	23.3
M	Caucasian	25	179.0	82.5	25.7
M	Black	29	183.0	86.2	25.7
F	Caucasian	48	169.0	74.1	25.9
F	Chinese	34	157.0	58.0	23.5
	Mean	37.2	170.7	73.9	25.3
	SD	12.6	10.0	9.6	2.1
¼ cup phase					
F	Caucasian	48	155.5	66.5	27.5
F	Caucasian	41	159.4	63.5	25.0
M	Chinese	54	170.4	78.7	27.1
M	Caucasian	24	178.0	83.4	26.3
M	Caucasian	24	183.5	78.2	23.2
M	Caucasian	57	176.6	79.8	25.6
M	Caucasian	56	170.7	79.5	27.3
F	Black	47	159.5	57.1	22.4
M	Black	28	175.1	75.0	24.5
F	Black	49	170.4	73.9	25.5
F	Black	37	162.7	68.1	25.7
M	Chinese	19	166.0	56.2	20.4
F	Caucasian	46	165.8	55.3	20.1
F	South East Asian	36	160.0	59.9	23.4
F	Caucasian	27	166.0	64.3	23.3
M	Caucasian	33	182.0	81.3	24.5
M	Caucasian	45	174.1	84.6	27.9
M	Caucasian	26	169.3	69.2	24.1
M	Caucasian	28	163.4	60.9	22.8
M	Caucasian	25	189.5	82.0	22.8
M	Caucasian	25	179.0	82.0	25.6
F	South Asian	23	150.5	47.0	20.8
M	Black	29	183.0	86.3	25.8
F	Caucasian	48	168.0	73.1	25.9
	Mean	36.5	169.9	71.1	24.5
	SD	12.0	9.7	11.0	2.2

**Table 4:** Incremental peak rise in blood glucose (mmol/L) and insulin after consumption of beans vs. control foods ^1^.

	Glucose, mmol/L				Insulin, pmol/L
Food	Corn	Macaroni	Potato	Rice	Potato
¼ cup phase	(*n* = 24)	(*n* = 16)	(*n* = 16)	(*n* = 16)	
Control	0.76 ± 0.07	0.93 ± 0.10	1.03 ± 0.09	1.09 ± 0.10	n/a
Black beans	0.25 ± 0.05 *	0.27 ± 0.07 *	0.26 ± 0.06 *	0.21 ± 0.06 *	n/a
Cranberry beans	0.36 ± 0.06 *	0.33 ± 0.06 *	0.35 ± 0.08 *	0.38 ± 0.07 *	n/a
Great northern beans	0.34 ± 0.03 *	0.31 ± 0.04 *	0.35 ± 0.04 *	0.36 ± 0.03 *	n/a
Kidney beans	0.31 ± 0.07 *	0.24 ± 0.04 *	0.32 ± 0.10 *	0.39 ± 0.09 *	n/a
Navy beans	0.31 ± 0.04 *	0.27 ± 0.04 *	0.34 ± 0.06 *	0.32 ± 0.05 *	n/a
Pinto beans	0.37 ± 0.04 *	0.35 ± 0.07 *	0.36 ± 0.06 *	0.41 ± 0.05 *	n/a
½ cup phase	(*n* = 18)	(*n* = 12)	(*n* = 12)	(*n* = 12)	(*n* = 12)
Control	1.67 ± 0.13	1.95 ± 0.18	2.05 ± 0.14	2.10 ± 0.23	8.6 [5.0, 16.7]
Black beans	0.62 ± 0.10 *	0.71 ± 0.13 *	0.54 ± 0.09 *	0.62 ± 0.14 *	2.8 [2.0, 4.7] ^†^
Cranberry beans	0.71 ± 0.07 *	0.77 ± 0.09 *	0.72 ± 0.09 *	0.66 ± 0.09 *	3.8 [1.8, 8.5]
Great northern beans	0.69 ± 0.11 *	0.73 ± 0.13 *	0.54 ± 0.13 *	0.81 ± 0.14 *	4.4 [2.9, 6.0]
Kidney beans	0.65 ± 0.11 *	0.66 ± 0.13 *	0.56 ± 0.08 *	0.73 ± 0.12 *	3.2 [1.6, 7.6]
Navy beans	0.47 ± 0.06 *	0.53 ± 0.08 *	0.41 ± 0.05 *	0.47 ± 0.09 *	2.1 [0.4, 5.8] ^††^
Pinto beans	0.92 ± 0.12 *	0.99 ± 0.16 *	0.84 ± 0.10 *	0.94 ± 0.19 *	4.3 [2.4, 7.0]

^1^ Values are means ± SEMs (normally distributed) or medians [interquartile ranges] (not normally distributed). Peak rise = [maximum value] − [fasting value] over a 2 h period. * Significantly different from control food by Dunnett’s test: Bonferroni-adjusted *p* < 0.001. ^†,††^ Significantly different from potato by Dunnett’s *p*-value: ^†^ *p* <0.05; ^††^ *p* <0.001. n/a = results not available.

**Table 5:** Glucose and insulin incremental areas under the curves and relative glycemic responses: beans vs. control food groups.

Food	Glucose								Insulin	
	Corn		Macaroni		Potato		Rice		Potato	
	iAUC, mmol × min/L	RGR, %	iAUC, mmol × min/L	RGR, %	iAUC, mmol × min/L	RGR, %	iAUC, mmol × min/L	RGR, %	iAUC, pmol × h/L	RIR, %
¼ cup phase	(*n* = 24)		(*n* = 16)		(*n* = 16)		(*n* = 16)			
Control	18.6 [13.2, 24.6]	100	30.1 [17.7, 41.1]	100	28.9 [22.4, 37.8]	100	42.0 [26.3, 60.7]	100	n/a	n/a
Black bean	10.5 [1.5, 18.5] *	**56**	9.3 [1.4, 16.8] **	**31**	11.2 [3.8, 22.8] *	**39**	10.5 [1.0, 16.7] **	**25**	n/a	n/a
Cranberry bean	12.0 [3.5, 18.8]	65	11.2 [7.4, 18.5] **	**37**	11.7 [1.3, 18.2] **	**40**	16.8 [10.1, 21.0] **	**40**	n/a	n/a
Great northern bean	14.8 [7.8, 21.9]	80	11.9 [6.8, 19.2] **	**40**	16.8 [10.9, 22.4]	**58**	16.8 [7.8, 22.4] **	**40**	n/a	n/a
Kidney bean	11.0 [2.5, 17.2] *	**59**	11.0 [2.1, 15.7] **	**37**	7.2 [2.2, 16.7] **	**25**	15.1 [7.3, 18.6] **	**36**	n/a	n/a
Navy bean	13.7 [8.5, 18.8]	74	10.6 [5.6, 15.7] **	**35**	13.8 [8.1, 20.7]	48	14.3 [11.6, 18.8] **	**34**	n/a	n/a
Pinto bean	15.8 [6.7, 27.1]	85	15.8 [2.8, 27.4] *	**52**	15.8 [8.3, 22.5]	55	15.9 [8.4, 27.4] **	**38**	n/a	n/a
Mean for 6 beans	14.1 [10.0, 20.3] ^†^	70 ^§^	12.7 [8.8, 17.5] ^†^	42 ^§^	13.0 [9.9, 21.9] ^†^	45 ^§^	15.4 [10.7, 20.9] ^†^	37 ^§^	n/a	n/a
½ cup phase	(*n* = 18)		(*n* = 12)		(*n* = 12)		(*n* = 12)		(*n* = 12)	
Control	54.6 ± 5.6	100	103.2 ± 11.6	100	67.4 ± 5.4 **	100	107.0 ± 10.8	100	39.0 ± 9.0	100
Black bean	29.0 ± 5.2 **	53	36.2 ± 6.6 **	35	26.7 ± 7.0 **	40	24.0 ± 5.2 **	22	27.8 ± 9.1	71
Cranberry bean	38.6 ± 4.1	71	41.0 ± 4.8 **	40	40.3 ± 5.4 *	60	34.4 ± 4.7 **	32	32.5 ± 11.3	83
Great northern bean	31.9 ± 5.2 **	**58**	33.9 ± 5.4 **	33	25.4 ± 7.0 **	38	36.3 ± 6.6 **	34	28.6 ± 7.2	73
Kidney bean	34.3 ± 5.7 *	63	33.9 ± 7.5 **	33	29.4 ± 5.6 **	44	39.6 ± 7.5 **	37	28.3 ± 6.9	73
Navy bean	26.0 ± 3.7 **	**48**	29.7 ± 4.8 **	29	20.5 ± 2.5 **	**30**	27.9 ± 5.6 **	26	17.0 ± 6.1	44
Pinto bean	43.5 ± 5.1	80	48.0 ± 6.0 **	47	40.3 ± 5.5 *	**60**	42.1 ± 7.2 **	39	31.6 ± 8.7	81
Mean for 6 beans ^§^	33.9 ± 3.3 ^†^	62 ^§^	37.1 ± 3.6 ^†^	36 ^§^	30.4 ± 3.6 ^†^	45 ^§^	34.1 ± 6.3 ^†^	32 ^§^	27.6 ± 6.3	71

Values are medians [25%ile, 75%ile] (for ¼ cup data which were not normally distributed) or means ± SEMs (for ½ cup data the distribution of which did not deviate significantly from normality). iAUC, incremental area under the curve; RGR, relative glycemic response (median or mean iAUC as % of control). RGR values in bold represent the minimum effective dose (MED). Significantly different from control food by Dunnett’s test: Bonferroni-adjusted *p*-value: * *p* < 0.05; ** *p* < 0.01. ^†^ Significantly different from control by paired *t*-test: Bonferroni-adjusted *p*-value < 0.01. ^§^ The mean/median iAUC for all six beans (e.g., 14.1 for ¼c beans vs. corn) expressed as a % of the mean/median iAUC for control. Each participant (*n* = 24 for ¼ cup and *n* = 18 for ½ cup) tested all six beans and corn and two of the other controls. n/a = results not available. The ¼ cup values for corn included 24 participants and macaroni, potato and rice included 16 participants, but these persons were not always the same, which is why the mean AUCs for the beans differ in the treatment columns.

**Table 6:** Ability of carbohydrate analysis to predict an effective reduction in glycemic response (eGR).

Test Foods (½ Cup Servings)	Control Foods (½ Cup Servings)											
	Corn			Macaroni			Potato			Rice		
	RGR	ivRGR	Out-Come	RGR	ivRGR	Out-Come	RGR	ivRGR	Out-Come	RGR	ivRGR	Out-Come
Black beans	53	53	Tp	35	37	Tp	40	48	Tp	22	32	Tp
Cranberry beans	(71)	84	Tn	40	55	Tp	60	75	Tp	32	46	Tp
Great northern beans	58	83	Fn	33	54	Tp	38	73	Tp	34	46	Tp
Kidney beans	63	86	Fn	33	56	Tp	44	76	Tp	37	47	Tp
Navy beans	48	67	Tp	29	45	Tp	30	59	Tp	26	38	Tp
Pinto beans	(80)	120	Tn	47	76	Tp	60	105	Fn	39	62	Tp
Rice *	56	60	Tp	(99)	92	Tn	72	67	Tp	-	-	-
Potato *	(82)	100	Tn	(74)	82	Tn	-	-	-	-	-	-
Macaroni *	48	73	Tp	-	-	-	-	-	-	-	-	-

eGR is defined as a statistically significant reduction in glucose iAUC of ≤20%. RGR is the observed relative glycemic response; values in brackets are not statistically significant. ivRGR: predicted relative glycemic response = SE + 100 × Te/Ce, where SE is the standard error of the y estimate from the multiple regression analysis (14.6) and Te and Ce are the y estimates calculated from the amounts of rapidly digested starch (RDS), slowly digested starch (SDS), resistant starch (RS) and available sugars (AS) present in the ½ cup portions of each test and control food, respectively, using the equation: 23.3 × RDS + 8.3 × SDS − 20.1 × RS + 39.5 × AS − 108.2. For example, the y estimates for black beans and corn, respectively, were 37.6% and 97.1% for an ivRGR of 14.6 + 38.7 = 53.3%. A value of ≤80% predicts an effective glucose reduction (eGR), i.e., the reduction in iAUC is both ≥20% and statistically significant. Outcomes: Tp = true positive (ERGauc predicted and ERGauc observed); Tn = true negative (no ERGauc predicted and no ERGauc observed); Fn = false negative (no ERGauc predicted but ERGauc observed). Of the *n* = 30 comparisons, *n* = 22 are Tp, *n* = 5 are Tn and *n* = 3 are Fn. * Post hoc comparison among the control foods based on paired *t*-tests; *n* = 12 for corn vs. rice, potato and macaroni; *n* = 6 for comparisons among rice, potato and macaroni. pRGR and observed RGR calculated as the lower iAUC as a % of the higher iAUC.

## Data Availability

The data presented in this study are available on request from the corresponding author. The data are not publicly available due to Crown copyright.
